# Exploitation of *Mycobacterium tuberculosis* Reporter Strains to Probe the Impact of Vaccination at Sites of Infection

**DOI:** 10.1371/journal.ppat.1004394

**Published:** 2014-09-18

**Authors:** Neelima Sukumar, Shumin Tan, Bree B. Aldridge, David G. Russell

**Affiliations:** 1 Cornell University, College of Veterinary Medicine, Department of Microbiology and Immunology, Ithaca, New York, United States of America; 2 Tufts University School of Medicine, Department of Molecular Biology and Microbiology, Boston, Massachusetts, United States of America; New Jersey Medical School, United States of America

## Abstract

*Mycobacterium tuberculosis* (Mtb) remains a major public health problem, with an effective vaccine continuing to prove elusive. Progress in vaccination strategies has been hampered by a lack of appreciation of the bacterium's response to dynamic changes in the host immune environment. Here, we utilize reporter Mtb strains that respond to specific host immune stresses such as hypoxia and nitric oxide (*hspX*′::GFP), and phagosomal maturation (*rv2390c*′::GFP), to investigate vaccine-induced alterations in the environmental niche during experimental murine infections. While vaccination undoubtedly decreased bacterial burden, we found that it also appeared to accelerate Mtb's adoption of a phenotype better equipped to survive in its host. We subsequently utilized a novel replication reporter strain of Mtb to demonstrate that, in addition to these alterations in host stress response, there is a decreased percentage of actively replicating Mtb in vaccinated hosts. This observation was supported by the differential sensitivity of recovered bacteria to the front-line drug isoniazid. Our study documents the natural history of the impact that vaccination has on Mtb's physiology and replication and highlights the value of reporter Mtb strains for probing heterogeneous Mtb populations in the context of a complex, whole animal model.

## Author Summary

*Mycobacterium tuberculosis* (Mtb) remains a serious challenge to global health, a situation that is exacerbated by emerging drug resistance, the paucity of new antibiotics, and the absence of an effective vaccine. Whilst there is no doubt that an anti-tuberculosis vaccine would have an extraordinary impact on the control of this disease, it is not clear if such a goal is achievable. One major deficiency in our knowledge is an appreciation of how the same protective immune response that limits bacterial growth is also responsible for driving adaptation of Mtb towards successful maintenance of long-term infection. We have used our panel of reporter Mtb strains that fluoresce under different environmental stresses for experimental infections of naïve and vaccinated, wild type and immune-deficient, mouse strains. In addition, we utilized a novel replication reporter strain to visualize the distribution of replicating versus non-replicating bacteria within infected lung tissue. The data provide novel insights into how the host immune response impacts bacterial fitness, physiology, and growth rates during the course of infection in naïve and vaccinated mice.

## Introduction

The potential impact that an effective vaccine against *Mycobacterium tuberculosis* (Mtb) would have in tuberculosis-endemic countries is undisputed, yet the route towards the development of such a vaccine has remained elusive. Most vaccine development strategies involve the use of recombinant Bacille Calmette-Guérin (BCG) expressing Mtb antigens, or BCG in combination with boosting the immune response with Mtb protein-adjuvant mixes or virally-encoded antigens [1]–[5]. None of these candidate vaccines, nor immunity following natural infection, effectively protects against infection or re-infection [6], [7]. Vaccine candidates that do show “efficacy” are able to reduce bacterial burden and pathology, but unfortunately do not prevent the infection establishing a stable “steady-state”, defined as a persistent, constant population of viable Mtb [8].

Much of the research in this area has focused on the nature of the immune response with respect to T-cell subsets, cytokine production, and the up-regulation of macrophage killing responses (see for example [2]–[4]). Few studies have attempted to elucidate the response of the pathogen to the host's immune environment, beyond the quantification of viable, persistent bacilli. Mtb causes a chronic infection that endures in its host and, in modern man, absent additional risk factors, only 5–10% of infected individuals will progress to active disease [9]–[12]. Not only has Mtb evolved to persist in the presence of a robust immune response, it actually requires that immune response to mediate the late stage damage necessary for completion of its life cycle and transmission to new hosts [9], [13], [14]. The basis of this ability appears to lie in the localized nature of the tissue response to infection, the granuloma. While an individual may have a robust systemic immune response, the consequences of that response, when translated through the granuloma, can have a range of different outcomes. Disease progression appears to be determined at the level of the individual granuloma, to such an extent that even in an individual who has active disease, granulomas in all states of development from cavitation to mineralization and resolution can be found [15], [16]. The bacterium's ability to maintain the infection even in the face of a strong immune response, through the localized manipulation of host tissue, represents an extraordinary challenge to successful vaccine development.

One of the areas that have been seriously neglected in understanding the basis of immune control of Mtb is the determination of the physiological status of the bacterium within these heterogeneous tissue environments. To address this, we have developed a range of bacterial stress and replication reporters that enable us to assess the fitness of Mtb in the context of its tissue environment. We have exploited these reporter strains to probe the infection sites in naïve and vaccinated murine models in both wild type and specific, immune-deficient mice. We found an apparent acceleration in Mtb's environmental niche progression in the presence of vaccination, as reflected in differences in reporter GFP fluorescence induction in vaccinated versus naïve hosts over a time course of infection. We also observed differences in the replication status and the isoniazid tolerance of Mtb recovered from vaccinated versus naïve host lungs. These results demonstrate the marked heterogeneity of the Mtb population during infection, and provide new insights into the dynamic nature of the host immune response as it impacts bacteria fitness and replication.

## Results and Discussion

### Intraperitoneal vaccination with heat-killed Mtb lowers bacterial burden and decreases lung pathology in mice

**Figure 1:**
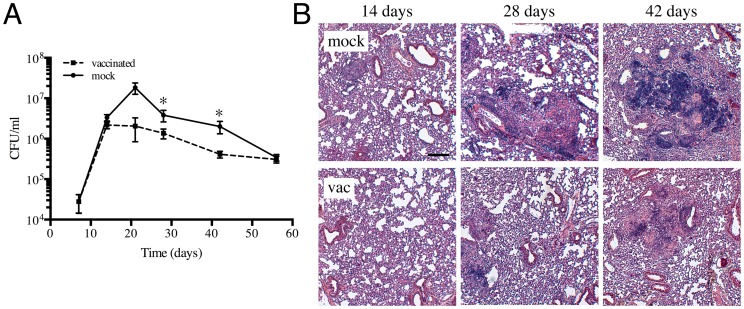
Characterization of a heat-killed Mtb vaccination model. (A) Kinetics of Mtb colonization in vaccinated or mock-treated mice lungs. C57BL/6J mice were intraperitoneally injected with 5×10^5^ CFUs of heat-killed Erdman or with sterile PBS (mock-treated). Four weeks post-vaccination, mice were challenged with 10^3^ CFUs of various reporter Mtb intranasally. At indicated time points post-challenge, mice were sacrificed and the bacterial burden in the lungs determined. The pooled data for each time point are shown as means ± SEM, from at least 3 animals per data point. * indicates p<0.05 (unpaired t-test). (B) Lung pathology of vaccinated and mock-treated mice. Lungs collected at indicated time points post-challenge were fixed in 4% paraformaldehyde and subjected to routine H&E staining. Scale bar 200 µm.

In order to examine how Mtb responds to dynamic changes in the host immune environment, we first established the appropriate vaccination and challenge parameters in a murine infection model. C57BL/6J mice were injected intraperitoneally with 5×10^5^ CFUs of heat-killed Erdman Mtb or PBS (mock-treated), prior to intranasal challenge four weeks later with 10^3^ Erdman Mtb. We chose to use heat-killed Erdman Mtb rather than BCG as the vaccination organism, to achieve the closest antigenic match between the vaccine and challenge bacteria. At various times post-challenge, mice were sacrificed and bacterial burden in the lungs determined. Although there was no, or only slight, differences in the CFUs recovered from the lungs of vaccinated versus mock-treated mice at 7 and 14 days post-challenge, lower bacterial burdens were observed in the immunized mice at 21, 28 and 42 days post-challenge (Figure 1A). At 56 days post-challenge, the bacterial burdens in the lungs of vaccinated versus mock-treated mice were comparable (Figure 1A). In accord with the CFU data, the lungs of Mtb-challenged immunized mice showed decreased cellular infiltration and tissue damage (Figure 1B). These results show that intraperitoneal vaccination with heat-killed Mtb leads to accelerated control of subsequent Mtb challenge, lowers bacterial burden, and reduces pathology associated with Mtb infection in mice lungs. The kinetics of infection progression in this experimental model of immune-mediated control provides a manipulable system for probing the impact of vaccine-induced immune responses on bacterial physiology and replication.

### The use of fluorescent reporter strains of Mtb to probe the host environment

Reporter Mtb strains serve as powerful tools to facilitate dynamic analyses of Mtb's response to its host environment at the level of the individual bacterium, within a complex whole animal model [17]. The reporter strains were constructed to express mCherry constitutively (*smyc*′::mCherry) to enable the visualization of all bacteria, in addition to the expression of GFP under the regulation of promoters of characterized responsiveness [17]. While we accept that the *in vivo* stresses examined are likely more complex than the *in vitro* induction conditions used to characterize the reporter constructs, the use of a diverse panel of reporter strains generates a coherent body of data that provide insights into the kinetics of the immune-mediated stresses experienced by Mtb during the course of infection.

**Figure 2:**
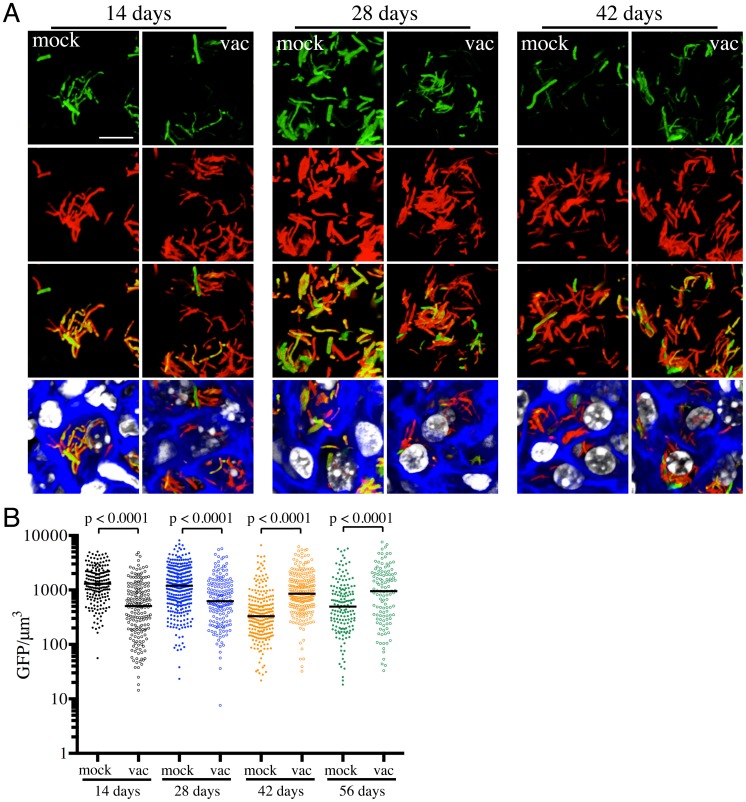
Differences in expression of *rv2390c*′::GFP in Mtb present in vaccinated versus mock-treated mice. Erdman(*rv2390c*′::GFP, *smyc*′::mCherry) was inoculated into vaccinated or mock-treated C57BL/6J WT mice for up to 56 days. (A) shows 3D confocal images from a 14, 28, or 42 day infection. All bacteria are marked in red (*smyc*′::mCherry), reporter signal is shown in green (*rv2390c*′::GFP), nuclei are marked in grayscale (DAPI), and phalloidin staining of f-actin is shown in blue. Scale bar 10 µm. (B) shows quantification of the GFP/µm^3^ signal for each bacterium measured from multiple 3D confocal images, at the indicated time points. Each point on the graph represents a bacterium or a tightly clustered group of bacteria (mock-treated – filled symbols, vaccinated – open symbols). Horizontal lines mark the median value for each group. p-values were obtained with a Mann-Whitney statistical test.

We had previously shown that pH and Cl^−^ act as synergistic cues for Mtb during host colonization, with changes in these environmental signals reflecting the immune status of the host cell [17]. To begin examining how vaccination impacts Mtb's physiology during establishment of infection, we exploited a pH and Cl^−^-responsive reporter strain of Mtb, Erdman(*rv2390c*′::GFP, *smyc*′::mCherry) [17], to challenge vaccinated and mock-treated mice. Mice were sacrificed at specific time points post-challenge, and the lungs fixed and examined by confocal microscopy. We observed relatively high *rv2390c*′::GFP induction in Mtb-infected mock-treated mice lungs on days 14 and 28 post-challenge, which decreased at later time-points (Figures 2A and 2B). In contrast, *rv2390c*′::GFP fluorescence was significantly lower in vaccinated mice at the earlier time points (14 and 28 days post-challenge), with this lower level fluorescence maintained through the course of infection (Figures 2A and 2B).

On initial examination, these results appear counter-intuitive. The data suggest that in mock-treated mice, Mtb initially encounters a high [Cl^−^]/low pH environment indicative of enhanced phagosomal maturation, which trends towards a lower [Cl^−^]/higher pH environment as the infection progresses. This indication of early stress during the establishment of infection is consistent with published studies in both macrophage and murine infections, which indicate that bacteria coming from broth culture have to re-align their physiology to be compatible with intracellular survival [18], [19]. During this adjustment period, rapidly growing organisms survive poorly [18], [19]. The confounding observation is that this early expression, or presence of *rv2390c*′::GFP, is absent in the vaccinated mice. The reduction in expression of *rv2390c*′::GFP in the presence of an acquired immune response, observed in the current study, may be best explained by either of two mechanistically-divergent scenarios. Firstly, while the adjustment in bacterial physiology required to support intracellular survival may come at a cost with respect to bacterial numbers, the re-alignment of bacterial physiology in the surviving bacteria may be accelerated by the presence of a pre-existing immune response to Mtb. Or, secondly, that bacilli expressing higher levels of *rv2390c′*::GFP may be the very bacteria that are killed most effectively by the pre-existing immune response. Although the underlying mechanisms may differ the outcome is the same; the accelerated generation of a bacterial population better equipped to survive in its host.

The experiments described above were conducted with “non-induced” bacteria. To test whether the state of induction of the reporter fluorophore impacted outcome, we challenged vaccinated or mock-treated mice with Erdman(*rv2390c*′::GFP, *smyc*′::mCherry) pre-induced for GFP expression by growth in the presence of 250 mM NaCl prior to infection. Pre-induction of *rv2390c*′::GFP did not alter the result, with Mtb in vaccinated mice once again exhibiting lower levels of *rv2390c′*::GFP fluorescence at early time points (Figure S1). There were no significant differences in the *rv2390c*′::GFP fluorescence levels of Mtb in mock-treated mice receiving either pre-induced or untreated Erdman(*rv2390c*′::GFP, *smyc*′::mCherry) (Figure S1). These results confirm that *rv2390c*′::GFP expression is under tight control and that even when pre-induced, the reduction in the level of expression, or relative numbers of *rv2390c*′::GFP-positive bacteria, is accelerated in the presence of a pre-existing immune response against Mtb.

### Vaccination alters the dynamics of nitric oxide production and/or hypoxia in the microenvironment surrounding Mtb

**Figure 3:**
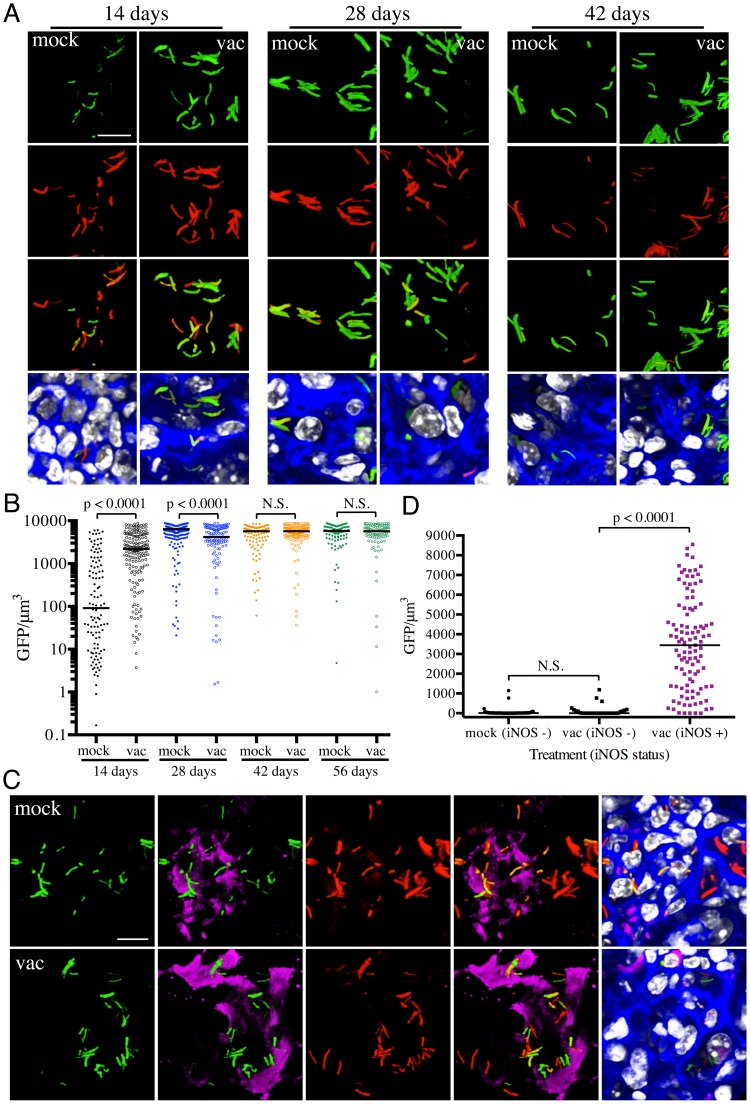
Differences in expression of *hspX*′::GFP in Mtb present in vaccinated versus mock-treated mice. (A and B) Higher *hspX*′::GFP induction in vaccinated mice at 14 days post-challenge. Erdman(*hspX*′::GFP, *smyc*′::mCherry) was inoculated into vaccinated or mock-treated C57BL/6J WT mice for up to 56 days. (A) shows 3D confocal images from a 14, 28, or 42 day infection. All bacteria are marked in red (*smyc*′::mCherry), reporter signal is shown in green (*hspX*′::GFP), nuclei are marked in grayscale (DAPI), and phalloidin staining of f-actin is shown in blue. Scale bar 10 µm. (B) shows quantification of the GFP/µm^3^ signal for each bacterium measured from multiple 3D confocal images, at the indicated time points. Each point on the graph represents a bacterium or a tightly clustered group of bacteria (mock-treated – filled symbols, vaccinated – open symbols). Horizontal lines mark the median value for each group. p-values were obtained with a Mann-Whitney statistical test. (C and D) *hspX*′::GFP-positive Mtb reside in iNOS-positive mouse lung regions. Erdman(*hspX*′::GFP, *smyc*′::mCherry) was inoculated into vaccinated or mock-treated C57BL/6J WT mice for 14 days. (C) shows 3D confocal images, with all bacteria marked in red (*smyc*′::mCherry), reporter signal shown in green (*hspX*′::GFP), iNOS stained in magenta, nuclei marked in grayscale (DAPI), and phalloidin staining of f-actin shown in blue. Scale bar 10 µm. (D) shows quantification of the bacterial GFP/µm^3^ signal, measured as in (B), in Mtb present in iNOS-positive versus negative regions. Horizontal lines mark the median value for each group. p-values were obtained with a Mann-Whitney statistical test.

The results with the *rv2390c*′::GFP reporter imply that, at least at the population level, the presence of an immune response accelerates Mtb's adoption of a physiological state conducive to maintenance of infection, albeit at a severe cost to bacterial survival. However, a pre-existing immune response also means the presence of robust CD4^+^ and CD8^+^ T-cell populations capable of activating macrophages to expresses anti-microbial activities such as reactive oxygen (ROI) and nitrogen (RNI) intermediates [8], [20]. *hspX* is one of the most highly up-regulated genes belonging to the *dos* regulon that is expressed in response to hypoxia and nitric oxide (NO) [21]–[24], and is known to be a useful correlate of the acquired immune response. We therefore utilized an Erdman(*hspX*′::GFP, *smyc*′::mCherry) reporter strain [17] to probe the kinetics of inducible nitric oxide synthase (iNOS) expression and the bacterium's response in both naïve and vaccinated mice. In marked contrast to *rv2390c*′::GFP expression, very low levels of *hspX*′::GFP fluorescence are seen initially, with extremely strong induction of *hspX*′::GFP fluorescence observed 4 weeks post-infection. These kinetics correlate with the establishment of an adaptive immune response, specifically IFNγ and iNOS production (Figures 3A and 3B) [8], [17]. Strikingly, we observed higher levels of *hspX*′::GFP fluorescence in Mtb present in vaccinated versus mock-treated mice at 14 days post-challenge (Figures 3A and 3B). *hspX*′::GFP fluorescence increased further at 28 days post-challenge in the vaccinated mice; however the levels of fluorescence induction in mock-treated mice now matched, and even surpassed, the levels observed in the vaccinated mice at this time point (Figures 3A and 3B), in accord with the establishment of an acquired immune response in the mock-treated mice and its effect on the *hspX*′::GFP reporter [8], [17]. These high levels of *hspX*′::GFP fluorescence were maintained at days 42 and 56 post-challenge in both groups of mice (Figures 3A and 3B).

To determine if the elevated levels of *hspX*′::GFP fluorescence in vaccinated mice correlated with both levels and distribution of iNOS expression, we stained the lung tissue sections with an antibody against murine iNOS. Consistent with data from previous studies [25], [26], the majority of the lung tissues analyzed from mock-treated mice at 14 days post-challenge were almost uniformly negative for iNOS. A rare iNOS-positive region in the mock-treated mice lung tissue is shown in Figure 3C. In contrast, iNOS-positive regions were frequent in lung tissue from Mtb-infected vaccinated mice at day 14 post-challenge (Figure S2). As reported previously, we observed greater *hspX*′::GFP fluorescence in Mtb residing in iNOS-positive versus iNOS-negative regions within the same lung tissue (Figures 3C and 3D) [17]. These data demonstrate, not surprisingly, that vaccination promotes an early onset of adaptive immune responses, and a correspondingly accelerated production of iNOS. These results provide the bacterium's perspective to the kinetics of development of an acquired immune response and the imposition of RNI-mediated stress at the level of the infected tissue.

### Utilization of select immune-deficient mice to demonstrate the specificity of Mtb's response to immune-mediated stress

To further validate the specificity of Mtb's responses to vaccination-induced alterations of the immune microenvironment, we probed the behavior of the fluorescent reporter strains in a select panel of immune-deficient mice. In the absence of IFNγ, Mtb-infected mice fail to activate macrophages or produce reactive nitrogen intermediates, resulting in unrestricted growth of Mtb and fatality [27], [28]. We have previously shown that there is significantly lower reporter Mtb GFP fluorescence in IFNγ^−/−^ mice infected with Erdman(*rv2390c*′::GFP, *smyc*′::mCherry) or Erdman(*hspX*′::GFP, *smyc*′::mCherry), consistent with the concept that Mtb infecting IFNγ^−/−^ hosts are present in less hostile microenvironments [17]. Here, we vaccinated IFNγ^−/−^ mice and challenged them with the different reporter Mtb strains, to determine the role of IFNγ in vaccination-induced modulation of Mtb's microenvironment. We restricted these studies to the first 28 days post-challenge, as mock-treated IFNγ^−/−^ mice succumb to Mtb infection early.

**Figure 4:**
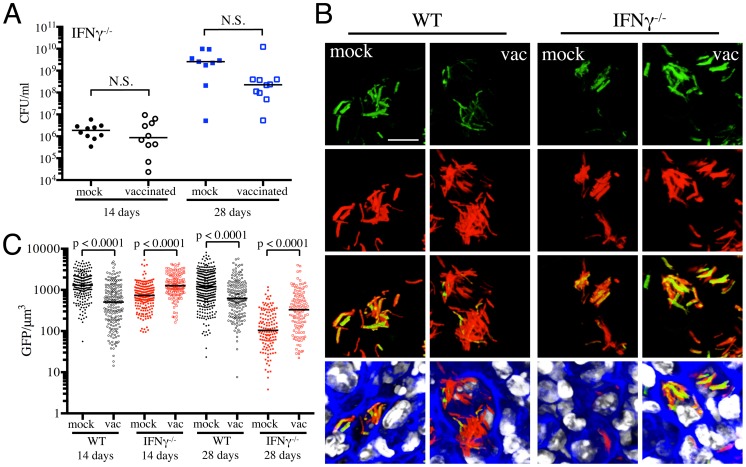
Dynamics of *rv2390c*′::GFP induced fluorescence in immune-deficient vaccinated versus mock-treated mice. (A) Bacterial burden in vaccinated and mock-treated IFNγ^−/−^ mice. Reporter Mtb strains were inoculated into vaccinated or mock-treated C57BL/6J IFNγ^−/−^ mice for up to 28 days. CFUs were determined by plating lung homogenates at 14 or 28 days post-challenge (mock-treated – filled symbols, vaccinated – open symbols). Horizontal lines mark the median value for each group. p-values were obtained with a Mann-Whitney statistical test. (B and C) *rv2390c*′::GFP induced fluorescence in vaccinated or mock-treated WT versus IFNγ^−/−^ mice. Erdman(*rv2390c*′::GFP, *smyc*′::mCherry) was inoculated into vaccinated or mock-treated C57BL/6J WT or IFNγ^−/−^ mice for up to 28 days. (B) shows 3D confocal images from a 14 day infection, with all bacteria marked in red (*smyc*′::mCherry), reporter signal shown in green (*rv2390c*′::GFP), nuclei marked in grayscale (DAPI), and phalloidin staining of f-actin shown in blue. Scale bar 10 µm. (C) shows quantification of the GFP/µm^3^ signal for each bacterium measured from multiple 3D confocal images, at the indicated time points. Each point on the graph represents a bacterium or a tightly clustered group of bacteria (mock-treated – filled symbols, vaccinated – open symbols; WT mice infections – black, IFNγ^−/−^ mice infections - red). Data for WT mice infections are as shown in Figure 2B. Horizontal lines mark the median value for each group. p-values were obtained with a Mann-Whitney statistical test.

At 14 days post-challenge we observed no differences in Mtb CFUs recovered from either mock-treated or vaccinated IFNγ^−/−^ mice, while at 28 days post-challenge, although not statistically significant, the vaccinated mice harbored lower bacterial CFUs as compared to the mock-treated IFNγ^−/−^ mice (Figure 4A). This is consistent with a previous study that showed decreased bacterial burden and enhanced median survival of Mtb-infected IFNγ^−/−^ mice on BCG vaccination [29]. In contrast to what was observed for WT mice, there was higher *rv2390c*′::GFP fluorescence in Mtb present in vaccinated versus mock-treated IFNγ^−/−^ mice at both time points tested (days 14 and 28 post-challenge) (Figures 4B and 4C). These data suggest that in the absence of an acquired immune response that includes IFNγ, the stress from components of the innate response, as indicated by *rv2390c*′::GFP expression, endures longer, or, alternatively, clearance of the *rv2390c′*::GFP-expressing bacteria is delayed.

**Figure 5:**
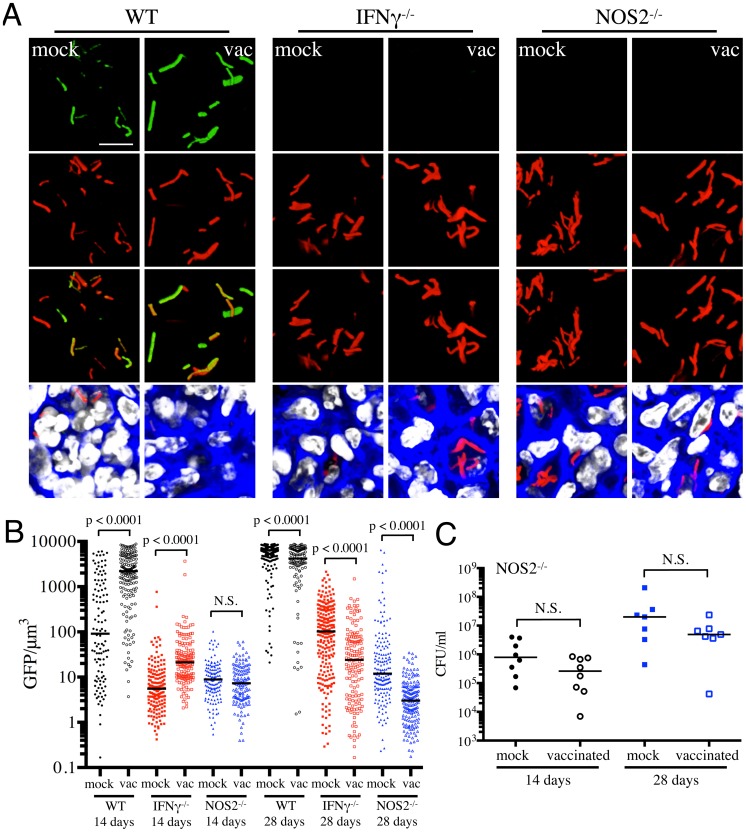
Dynamics of *hspX*′::GFP induced fluorescence in immune-deficient vaccinated versus mock-treated mice. (A and B) *hspX*′::GFP induced fluorescence in vaccinated or mock-treated IFNγ^−/−^ and NOS2^−/−^ mice. Erdman(*hspX*′::GFP, *smyc*′::mCherry) was inoculated into vaccinated or mock-treated C57BL/6J WT, IFNγ^−/−^, or NOS2^−/−^ mice for up to 28 days. (A) shows 3D confocal images from a 14 day infection, with all bacteria marked in red (*smyc*′::mCherry), reporter signal shown in green (*hspX*′::GFP), nuclei marked in grayscale (DAPI), and phalloidin staining of f-actin shown in blue. Scale bar 10 µm. (B) shows quantification of the GFP/µm^3^ signal for each bacterium measured from multiple 3D confocal images, at the indicated time points. Each point on the graph represents a bacterium or a tightly clustered group of bacteria (mock-treated – filled symbols, vaccinated – open symbols; WT mice infections – black, IFNγ^−/−^ mice infections – red, NOS2^−/−^ mice infections - blue). Data for WT mice infections are as shown in Figure 3B. Horizontal lines mark the median value for each group. p-values were obtained with a Mann-Whitney statistical test. (C) Bacterial burden in vaccinated and mock-treated NOS2^−/−^ mice. Erdman(*hspX*′::GFP, *smyc*′::mCherry) was inoculated into vaccinated or mock-treated NOS2^−/−^ mice for up to 28 days. CFUs were determined by plating lung homogenates at 14 or 28 days post-challenge (mock-treated – filled symbols, vaccinated – open symbols). Horizontal lines mark the median value for each group. p-values were obtained with a Mann-Whitney statistical test.

With Erdman(*hspX*′::GFP, *smyc*′::mCherry), we observed significantly lower levels of reporter Mtb GFP fluorescence in IFNγ^−/−^ mice as compared to WT mice, irrespective of treatment (Figures 5A and 5B), consistent with iNOS expression being the major inducer of *hspX*′::GFP expression. Despite this clear difference in the absolute levels of induction, the trends in *hspX*′::GFP fluorescence between the vaccinated and the mock-treated mice were similar in both WT and IFNγ^−/−^ mice, with higher GFP signal in Mtb present in vaccinated versus mock-treated IFNγ^−/−^ mice at 14 days post-challenge (Figures 5A and 5B). These data indicate that the induction of fluorescence in these reporter strains should not be viewed as a consequence solely of expression of enzymes such as iNOS.

To probe this phenomenon more deeply, we utilized NOS2^−/−^ mice to determine the dynamics of *hspX*′::GFP expression in the absence of iNOS. We observed a slightly lower bacterial burden in vaccinated versus mock-treated NOS2^−/−^ mice, although the differences were not statistically significant (Figure 5C). Similar to IFNγ^−/−^ mice, *hspX*′::GFP fluorescence was present at very low levels in Mtb present in NOS2^−/−^ mice (Figures 5A and 5B). While no differences in Mtb *hspX*′::GFP reporter fluorescence was observed at 14 days post-challenge in NOS2^−/−^ mice, lower *hspX*′::GFP fluorescence was seen in vaccinated versus mock-treated NOS2^−/−^ mice at 28 days post-challenge (Figures 5A and 5B). The results with the NOS2^−/−^ mice confirm that, while other factors impact this bacterial reporter strain, NO is the major driver of *hspX* expression in Mtb within the murine model.

### Vaccination decreases the percentage of actively replicating Mtb

**Figure 6:**
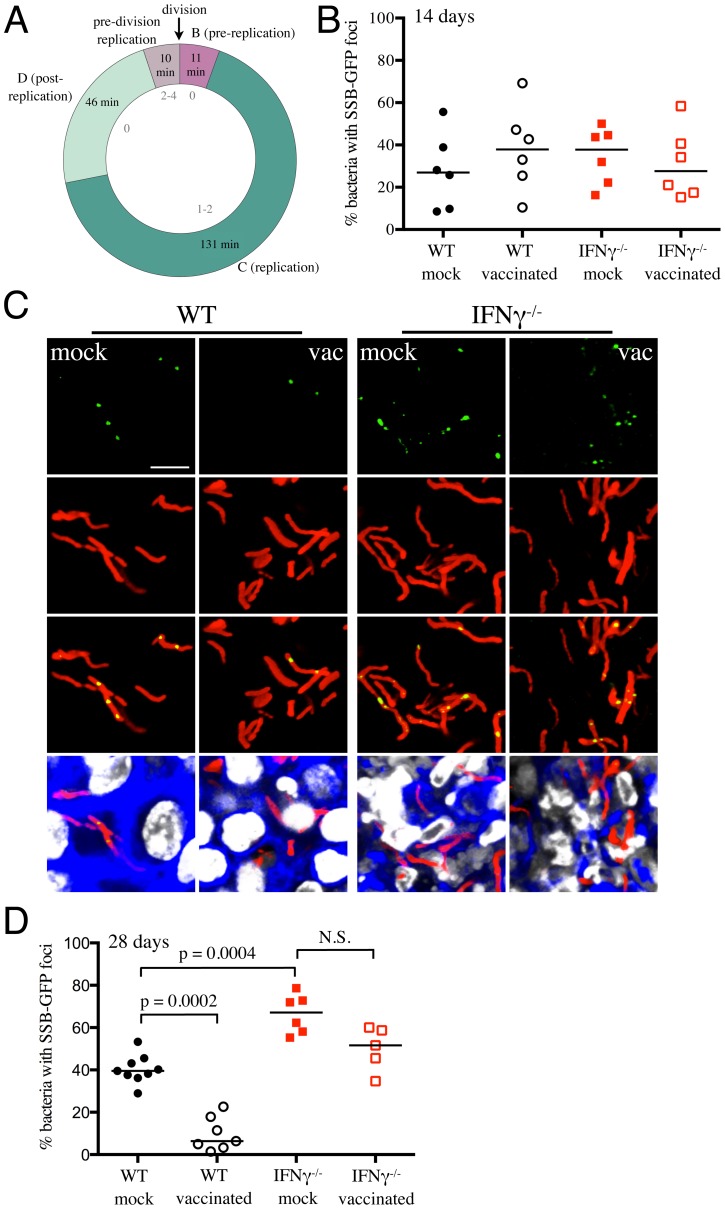
Mtb replication in vaccinated versus mock-treated mice. (A) SSB-GFP defines cell cycle timing in *M. smegmatis*. SSB-GFP expressing *M. smegmatis* cells were imaged every 15 minutes while growing in a microfluidic device (as described in [38]) in 7H9 supplemented with 10% ADC, 0.2% glycerol, 0.05% Tween 80, and 50 µg/ml hygromycin B. The medium was further supplemented with 2% DMSO and 0.0625 µg/ml FM4-64 FX to visualize septal membranes. The number of SSB-GFP foci (gray, inside of the chart) indicates the status of DNA replication in single cells. The average time in each period of the cell cycle is indicated (n = 122). B (G1) is the period after division but before initiation of DNA replication, C (S) is the period of DNA replication, and D (G2) is the period between the termination of DNA replication and division (defined as cell wall pinching or v-snapping). Some cells begin a new round of replication before division; daughters of these cells do not spend time in B. Standard deviations are 19, 38, 26, and 13 minutes for B, C, D, and pre-division replication, respectively. (B–D) Erdman(SSB-GFP, *smyc*′::mCherry) was inoculated into vaccinated or mock-treated C57BL/6J WT or IFNγ^−/−^ mice for up to 28 days. (B) shows the percentage of Mtb displaying SSB-GFP foci for each mouse, measured from multiple 3D confocal images, at 14 days post-challenge. Each point on the graph represents a mouse (mock-treated – filled symbols, vaccinated – open symbols; WT mice infections – black, IFNγ^−/−^ mice infections – red). Horizontal lines mark the median value for each group. (C) shows 3D confocal images from a 28 day infection, with all bacteria marked in red (*smyc*′::mCherry), reporter signal shown in green (SSB-GFP), nuclei marked in grayscale (DAPI), and phalloidin staining of f-actin shown in blue. For clarity of foci visualization, SSB-GFP signal is shown in extended focus, overlaid on the 3D image. Scale bar 10 µm. (D) shows the percentage of Mtb displaying SSB-GFP signal for each mouse, measured as in (B), at 28 days post-challenge. Each point on the graph represents a mouse (mock-treated – filled symbols, vaccinated – open symbols; WT mice infections – black, IFNγ^−/−^ mice infections – red). Horizontal lines mark the median value for each group. p-values were obtained with a Mann-Whitney statistical test.

Figure 1 clearly demonstrates that vaccination reduces the number of viable bacteria present in the mouse when the infection first transitions to “steady state”. However, the dynamic nature of this population is not well understood. To what extent does this represent overlapping curves of replication versus death, and to what extent does this represent limited bacterial replication? While this question has been studied at the population level, the degree of heterogeneity within that population is unknown. To extend the data from the reporter strains, we sought to determine the effect of vaccination on Mtb's replication status, at the level of the individual bacillus. For this purpose we constructed a replication reporter, Erdman(SSB-GFP, *smyc*′::mCherry), consisting of a fusion of single stranded binding protein (SSB) to GFP, present on a replicating plasmid containing a constitutively expressed mCherry. In *Escherichia coli*, fluorescent tagging of components of the replisome, such as SSB, marks DNA replication initiation by the appearance of a replisome focus, which can be tracked through DNA replication, with foci disappearance upon termination of DNA replication [30]. SSB-fluorophore fusions have also been validated extensively as a marker of active DNA replication in *Bacillus subtilis* [31], [32]. We established the dynamics of SSB-GFP expression using live-cell time-lapse microscopy of *Mycobacterium smegmatis* expressing the replication reporter construct. As anticipated, the SSB-GFP reporter defined cell cycle timing, with SSB-GFP foci present for periods of DNA replication (Figure 6A and Video S1). As in *E. coli* and *B. subtilis*, we observed that SSB-GFP foci disappeared when the bacteria stopped growing (such as during stationary phase). Consistent with the duration of the SSB-GFP foci in replicating *M. smegmatis*, we also observed that 76%–80% of log-phase broth cultures of Mtb exhibited SSB-GFP foci. CFU enumeration provides population-based information on the numbers of surviving Mtb within the host, but does not provide insight into population heterogeneity in the context of Mtb's tissue environment. We note that the lack of SSB-GFP foci in a cell may indicate either a non-growing bacterium, or mark a growing cell not in the active stage of DNA replication. Time point-based experiments with the Erdman(SSB-GFP, *smyc*′::mCherry) reporter strain will thus present snapshots that allow us to visualize the heterogeneity in replication status within the bacterial population, as well as to quantify active DNA replication at the level of the individual bacterium.

WT and IFNγ^−/−^ mice were vaccinated or mock-treated, 4 weeks before challenge with Erdman(SSB-GFP, *smyc*′::mCherry). At 14 days post-challenge, there was marked heterogeneity in the percentage of actively replicating Mtb even within treatment groups, and no statistically significant differences between Mtb in vaccinated and mock-treated WT and IFNγ^−/−^ mice were observed (Figure 6B). However, distinct differences in the replication status of Mtb between experimental groups were seen at 28 days post-challenge (Figures 6C and 6D). Confocal microscopy revealed significantly fewer Mtb exhibiting SSB-GFP foci in vaccinated versus mock-treated WT mice (6.4% versus 39.5% respectively, p = 0.0002) (Figures 6C and 6D). Consistent with the increased growth of Mtb in IFNγ^−/−^ mice at 28 days post-challenge, we observed a greater number of bacteria positive for SSB-GFP foci in these mice (67.1% versus 39.5% for mock-treated WT versus IFNγ^−/−^ mice, p = 0.0004) (Figures 6C and 6D). Similar to WT mice however, a lower percentage of Mtb present in vaccinated IFNγ^−/−^ mice exhibited SSB-GFP foci versus mock-treated IFNγ^−/−^ mice, although we did not have statistical significance in this case (51.6% versus 67.1%, p = 0.052) (Figures 6C and 6D). These results indicate that vaccination leads to a decreased percentage of actively-replicating Mtb in the host.

### Differential drug susceptibility of Mtb supports the growth profiles indicated by the replication reporter

The replication status of Mtb is reflected in its differential sensitivity to drugs. Isoniazid (INH) inhibits mycolic acid synthesis, which is required for cell wall synthesis and shows preferential activity against actively replicating organisms, while rifampicin (Rif) inhibits DNA-dependent RNA polymerase and shows more similar levels of activity against replicating and static organisms [33], [34]. Comparative differences in the tolerance of Mtb to INH and Rif are commonly utilized to assess the growth state of Mtb [35], [36].

**Figure 7:**
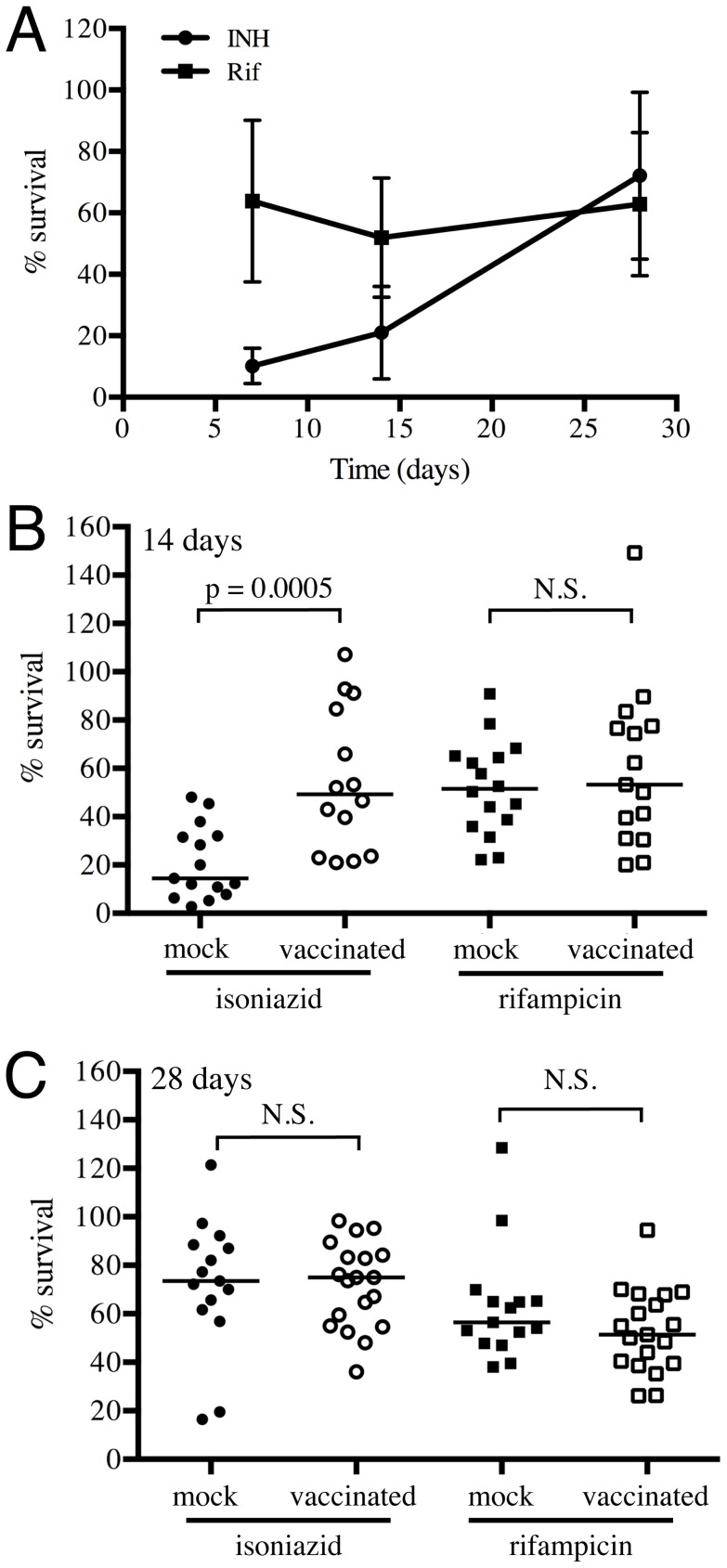
Mtb in vaccinated lungs display higher tolerance to isoniazid but not rifampicin. (A) Kinetics of tolerance of Mtb to INH and Rif in mock-treated mice. Erdman reporter strains were inoculated into C57BL/6J WT mice for up to 28 days. Lung homogenates collected at days 7, 14 and 28 post-challenge were subjected to treatment with 0.4 µg/ml INH or Rif, or DMSO as a control, for 18–20 hours, and the percentage of Mtb surviving INH or Rif treatment determined. (B and C) Erdman reporter strains were inoculated into vaccinated or mock-treated C57BL/6J WT mice for up to 28 days. Lung homogenates collected from vaccinated or mock-treated mice at days 14 (B) or 28 (C) post-challenge were subjected to treatment with 0.4 µg/ml INH or Rif, or DMSO as a control, for 18–20 hours, and the percentage of Mtb surviving INH or Rif treatment determined. Each point on the graph represents a mouse (mock-treated – filled symbols, vaccinated – open symbols; circle – INH treatment, square – Rif treatment). Horizontal lines mark the median value for each group. p-values were obtained with a Mann-Whitney statistical test.

We collected lung homogenates from either vaccinated or mock-treated mice at various time-points and incubated them with 0.4 µg/ml of INH or Rif, or DMSO as a control, for 24 hours, before plating to determine CFUs of surviving bacteria. We observed a gradual increase in INH tolerance of Mtb recovered from mock-treated mice as the infection progressed from 7 to 28 days post-challenge, while Rif tolerance remained unchanged (Figure 7A). Most notably, Mtb recovered at 14 days post-challenge from vaccinated mice displayed greater tolerance to INH but not Rif (Figure 7B); however, this differential drug sensitivity was lost by day 28 post-challenge (Figure 7C). These results suggest that vaccination, through the accelerated acquisition of an acquired immune response, leads to immune-mediated control of bacterial replication; a finding consistent with the data generated by the Erdman(SSB-GFP, *smyc*′::mCherry) replication reporter strain.

### Conclusions

While there is an extensive body of literature exploring the nature of the immune response to Mtb from the host's perspective, few studies, beyond those analyzing the phenotypes of Mtb mutants, have attempted to address the infection process from the perspective of the bacterium. And yet this infection represents an exquisite balance between persistence and disease in which both host and pathogen play equally critical roles. While it is true that the mouse is not ideal as a model for the progression of human disease, it is an important tool for validation of new reagents and probes such as the reporter strains characterized in this current study.

Data in this study illustrate the dynamics of Mtb fitness and adaptation in a vaccinated versus naïve host immune environment. While vaccination with heat-killed Mtb clearly reduced the bacterial burden at onset of immune-mediated control, it also appeared to either accelerate the resolution of the innate immune stresses experienced by Mtb early in infection, or accelerate “purification” of the population and the death of those bacilli least physiologically-adapted to survive. The results with the SSB-GFP replication reporter and tests on INH and Rif tolerance also suggest a hastened onset of a more persistent stage of Mtb colonization in the presence of vaccination. These findings raise questions about the feasibility of developing a sterilizing vaccine, and on the impact of a non-sterilizing vaccination on the progression of a subsequent Mtb infection. Is sterilizing immunity via vaccination achievable considering Mtb's exquisite ability to resist clearance in the face of an activated immune system? Are there possible adverse impacts on subsequent Mtb infection that may be prompted by a non-sterilizing vaccination? We feel that these are fundamental issues that should be considered in the development of Mtb vaccination and treatment strategies.

Our experiments further demonstrate the utility of reporter Mtb strains for the *in vivo* analysis of host-pathogen interactions [17]. In addition to reporter Mtb strains responsive to defined host environmental cues such as pH and NO [17], we have validated a novel Mtb replication reporter, SSB-GFP, which enable determination of Mtb's replication status at the level of the individual microbe and its site of infection. This is particularly significant as other methods for assessing Mtb replication, such as the use of a “clock” plasmid that is lost from replicating bacilli at a fixed rate [18], allows monitoring of Mtb replication at the population level, but cannot place replication status in the spatial context of Mtb's microenvironment. Coupling the use of these reporters with confocal microscopy and careful quantification of individual bacteria allowed Mtb's heterogeneous response to the dynamic host immune system to be evaluated. Studies using rabbit or macaque models of Mtb infection have previously reported the lesion to lesion heterogeneity that exists within the lungs of a single animal, with the progression and fate of each lesion being independent [15], [16]. Our study shows that heterogeneity during Mtb infection is present not just at the level of the host tissue, but is also a function across the population of infecting bacteria. The degree to which this bacterial heterogeneity “prepares” Mtb to exploit the diverse and evolving tissue environments of the host is a challenging question but one rendered more accessible through the reporter strains detailed here. We believe that this marked heterogeneity is biologically significant and propose that further studies exploiting such reporter tools in appropriate animal models will yield new insights into the factors that tip the host/pathogen balance.

## Materials and Methods

### Ethics statement

Animal procedures were performed in strict accordance with the National Institutes of Health “Guide for the Care and Use of Laboratory Animals”, and with the approval of the Institutional Animal Care and Use Committee of Cornell University (protocol number 2011-0086). All efforts were made to minimize suffering.

### Fluorescent reporter Mtb strains

Erdman(*smyc*′::mCherry) was generated by transforming Erdman WT with the replicating plasmid pCherry3, and selecting for transformants on 7H10 agar containing 50 µg/ml hygromycin B [37]. Erdman(*rv2390c*′::GFP, *smyc*′::mCherry) and Erdman(*hspX*′::GFP, *smyc*′::mCherry) have been previously described [17]. To generate Erdman(SSB-GFP, *smyc*′::mCherry), a 334 bp region immediately upstream of *ssb* together with the *ssb* open reading frame (excluding the stop codon) was PCR amplified from Mtb genomic DNA. This was fused to *gfpmut2* amplified from the vector pSE100, by overlapping PCR. An 11 amino acid linker region (SAGSAAGSGEF) was included between the *ssb* and *gfpmut2* open reading frames, to help prevent interference of SSB function by GFP [30]. This construct was subcloned into pCherry3 and transformed into Erdman Mtb. Selection for transformants was carried out on 7H10 agar containing 50 µg/ml hygromycin B. The reporter Mtb strains were grown to log phase in 7H9 broth supplemented with 50 µg/ml hygromycin B, aliquoted in 10% glycerol, and stored at −80°C until use. For Mtb infections in mice, aliquots were thawed, passed 3–5 times through a tuberculin syringe, and diluted to the required CFUs in sterile phosphate buffered saline (PBS) containing 0.05% Tween 80. For Cl^−^ induction of Erdman(*rv2390c*′::GFP, *smyc*′::mCherry), the reporter strain was grown to log phase and seeded at an OD_600_ of 0.05 in 7H9 media containing 250 mM NaCl, buffered at pH 7 with 100 mM MOPS. The induction was allowed to continue for 6 days, after which an aliquot was diluted in PBS containing 0.05% Tween 80 to the necessary CFUs for mice infections.

### Mice vaccination and infections

6–8 week old C57BL/6J, B6.129S7-*Ifng^tm1Ts^*/J (IFNγ^−/−^) and B6.129P2-*Nos2^tm1Lau^*/J (NOS2^−/−^) mice were obtained from Jackson Laboratories. Mice were intraperitoneally injected with 5×10^5^ CFUs of heat-killed Erdman, or as control, 100 µl of PBS. Four weeks post-vaccination, mice were intranasally challenged with approximately 1000 CFUs of different Erdman reporter strains in 25 µl of PBS containing 0.05% Tween 80. The inoculum dosage was confirmed by plating different dilutions on 7H10 plates supplemented with 50 µg/ml hygromycin B. At various times post-challenge, mice were sacrificed and the left lung lobe and the accessory lobe of the right lung homogenized in PBS containing 0.05% Tween 80. Bacterial loads were determined by plating serial dilutions of the homogenates on 7H10 agar +/−50 µg/ml hygromycin B. Plates were incubated at 37°C and colonies enumerated 3–4 weeks after. The other lobes of the right lung were harvested, fixed overnight in 4% paraformaldehyde, and transferred into sterile PBS until examination. All animals were maintained in a specific pathogen-free Biosafety level-3 facility.

### Lung histopathology

Paraformaldehyde fixed lung lobes were subjected to routine hematoxylin and eosin staining by the Cornell Histology Laboratory. Stained sections were imaged using a Zeiss Axio Imager M1 equipped with an AxioCam HRc camera.

### Confocal microscopy and quantification of reporter expression

Processing of lung tissues for confocal microscopy and reporter Mtb expression quantification was carried out as previously described [17]. In brief, sections of fixed lung tissues were stained with DAPI and Alexa Fluor 647 conjugated phalloidin (Invitrogen), mounted with Vectashield mounting medium (Vector Labs), and imaged using a Leica SP5 confocal microscope. Z-stacks were reconstructed into 3D using Volocity software (PerkinElmer), and Mtb GFP expression quantified by simultaneously measuring the voxel volume of each bacterium, via the mCherry channel, and the corresponding sum of the GFP signal for that bacterium. Images for the different reporter strains were taken with the GFP setting fixed within experimental sets and time points to allow comparison. At least 100 bacteria were quantified for each condition. Quantification of the SSB-GFP reporter was accomplished by counting the number of bacteria with and without SSB-GFP replication foci, and calculating the total percentage of bacteria presenting one or more SSB-GFP foci for all regions imaged for each animal. Lung sections from at least 5–9 mice per treatment per time point were imaged, and at least three images from different regions of each lung section were used for quantification. Statistical analyses were carried out by a non-parametric Mann-Whitney test.

Staining of iNOS was also carried out as previously described [17]. In brief, lung sections were permeabilized and blocked for 1 hour at room temperature with blocking buffer (PBS +3% Bovine Serum Albumin +0.1% Triton X-100). Sections were incubated with rabbit anti-iNOS (BD Transduction Labs) at a dilution of 1∶100, overnight at 4°C. After washing with blocking buffer, the sections were incubated with Alexa Fluor 514 goat anti-rabbit (1∶200) (Invitrogen), DAPI (1∶500), and Alexa Fluor 647 conjugated phalloidin (1∶50) (Invitrogen) at room temperature for 2 hours. The samples were then washed and mounted with Vectashield mounting medium and analyzed by confocal microscopy.

### Live-cell time-lapse imaging of *M. smegmatis*

*M. smegmatis* strain mc^2^155 was transformed with a SSB-GFP replicating plasmid as described above. *M. smegmatis* cells expressing SSB-GFP were grown and imaged in a microfluidic device as previously described [38]. Images were acquired every 15 minutes for 18–24 hours, using a PersonalDV Delta Vision widefield microscope equipped with hardware-based autofocus and an EM-CCD camera. Low-light imaging conditions were used to prevent phototoxicity and DNA damage. To visualize septal membranes, the medium was supplemented with 2% DMSO and 0.0625 µg/ml FM4-64 FX (Invitrogen). Newly divided cells generally did not have an SSB-GFP focus, and we labeled this the pre-replication period. The period where there were 1–2 SSB-GFP foci was defined as the replication stage, and the subsequent period where foci were absent was labeled as the post-replication stage. In some cells, SSB-GFP foci appeared after D but before septation, and this stage was labeled as “pre-division replication. Images were processed and analyzed using ImageJ, ObjectJ, and custom software written in MATLAB R2013a.

### Isoniazid and rifampicin assays

Lung homogenates (100 µl) obtained from Mtb infected vaccinated or mock-treated mice harvested at different time points were incubated with 0.04 µg/ml isoniazid (INH), rifampicin (Rif), or DMSO as a control, in a total volume of 200 µl in 7H9 medium in 96 well plates. The plates were incubated for 18–20 hours at 37°C, after which bacterial CFUs were enumerated by plating different dilutions on 7H10 plates. The percentage of surviving bacteria was calculated by dividing the CFUs obtained after INH or Rif treatment by CFUs obtained after control DMSO treatment, multiplied by 100.

## Supporting Information

Figure S1 **Pre-induction of** ***rv2390c*** **′::GFP signal does not alter** ***in vivo rv2390c′*** **::GFP fluorescence.** Erdman(*rv2390c*′::GFP, *smyc*′::mCherry) was grown in broth culture +/−250 mM NaCl, pH 7, for 6 days prior to inoculation into vaccinated or mock-treated C57BL/6J WT mice for 14 days. (A) shows 3D confocal images, with all bacteria marked in red (*smyc*′::mCherry), reporter signal shown in green (*rv2390c*′::GFP), nuclei marked in grayscale (DAPI), and phalloidin staining of f-actin shown in blue. Scale bar 10 µm. (B) shows quantification of the GFP/µm^3^ signal for each bacterium measured from multiple 3D confocal images, at the indicated time points. Each point on the graph represents a bacterium or a tightly clustered group of bacteria (mock-treated – filled symbols, vaccinated – open symbols; untreated Mtb inoculum – black, pre-induced Mtb inoculum - red). Horizontal lines mark the median value for each group. p-values were obtained with a Mann-Whitney statistical test.
(TIF)

Figure S2 **iNOS-positive regions are more frequent in Mtb-infected vaccinated mice lung tissues at day 14 post-challenge.** Erdman(*hspX*′::GFP, *smyc*′::mCherry) was inoculated into vaccinated or mock-treated C57BL/6J WT mice for 14 days. Two sets of 3D confocal images are shown for each treatment condition, with all bacteria marked in red (*smyc*′::mCherry), reporter signal shown in green (*hspX*′::GFP), iNOS stained in magenta, nuclei marked in grayscale (DAPI), and phalloidin staining of f-actin shown in blue. Scale bar 20 µm.
(TIF)

Video S1 **Live fluorescence time-lapse of SSB-GFP expressing** ***M. smegmatis*** **growth in a microfluidic device.** *M. smegmatis* expressing SSB-GFP (green) was grown in a microfluidic device, in media supplemented with FM4-64 FX for visualization of septal membranes (red). Images were collected every 15 minutes for 12.75 hours. The movie is compressed into 10 seconds (4590× speed).
(MOV)
