# Supplementary figures and images for: Exploitation of Mycobacterium tuberculosis Reporter Strains to Probe the Impact of Vaccination at Sites of Infection
Source: PLoS Pathog. 2014 Sep 18;10(9):e1004394. doi: 10.1371/journal.ppat.1004394 (PMC4169503; doi:10.1371/journal.ppat.1004394)

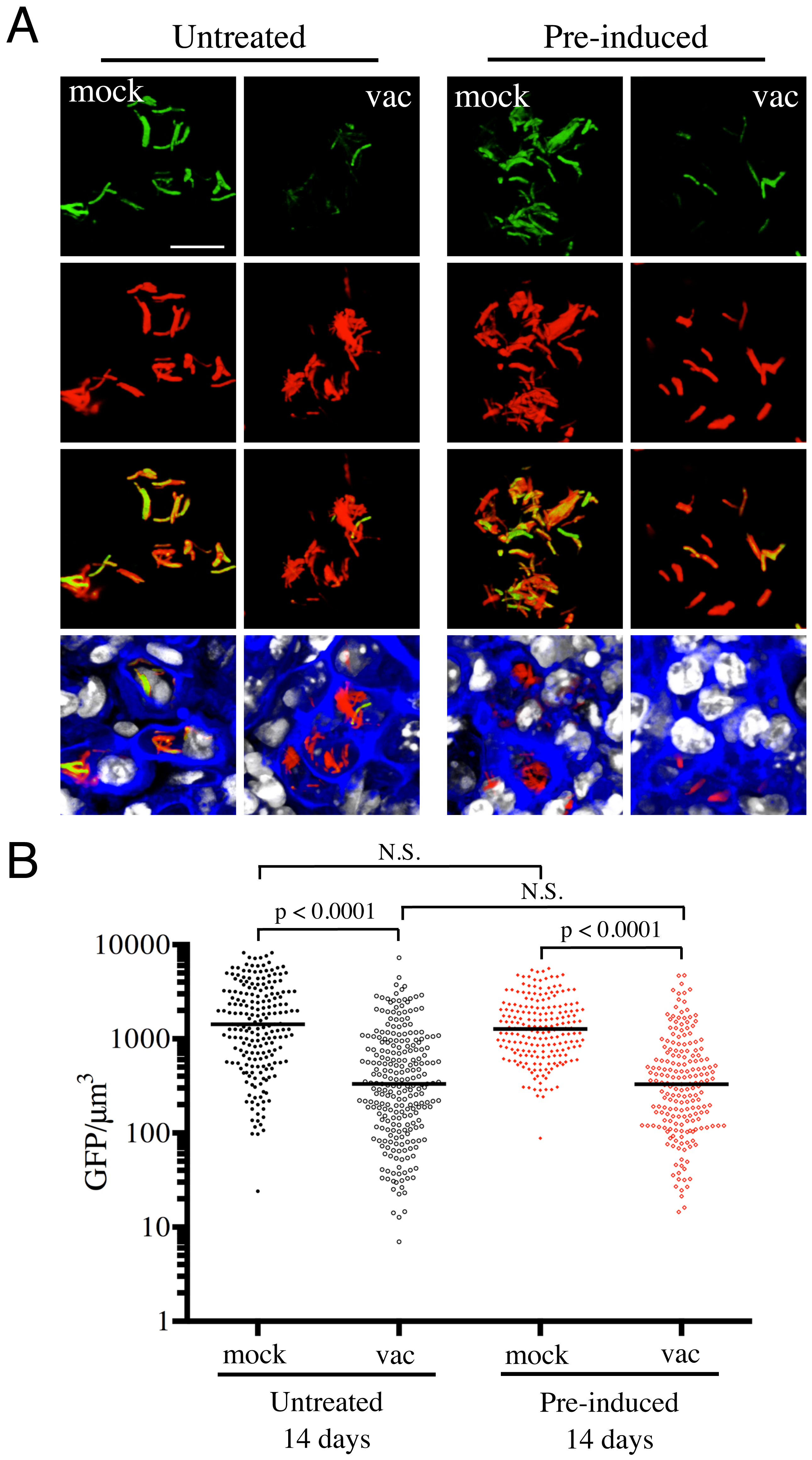

Supplement: Figure S1 — Pre-induction of rv2390c ′::GFP signal does not alter in vivo rv2390c′ ::GFP fluorescence. Erdman(rv2390c′::GFP, smyc′::mCherry) was grown in broth culture +/−250 mM NaCl, pH 7, for 6 days prior to inoculation into vaccinated or mock-treated C57BL/6J WT mice for 14 days. (A) shows 3D confocal images, with all bacteria marked in red (smyc′::mCherry), reporter signal shown in green (rv2390c′::GFP), nuclei marked in grayscale (DAPI), and phalloidin staining of f-actin shown in blue. Scale bar 10 µm. (B) shows quantification of the GFP/µm3 signal for each bacterium measured from multiple 3D confocal images, at the indicated time points. Each point on the graph represents a bacterium or a tightly clustered group of bacteria (mock-treated – filled symbols, vaccinated – open symbols; untreated Mtb inoculum – black, pre-induced Mtb inoculum - red). Horizontal lines mark the median value for each group. p-values were obtained with a Mann-Whitney statistical test. (TIF) [file ppat.1004394.s001.tif]

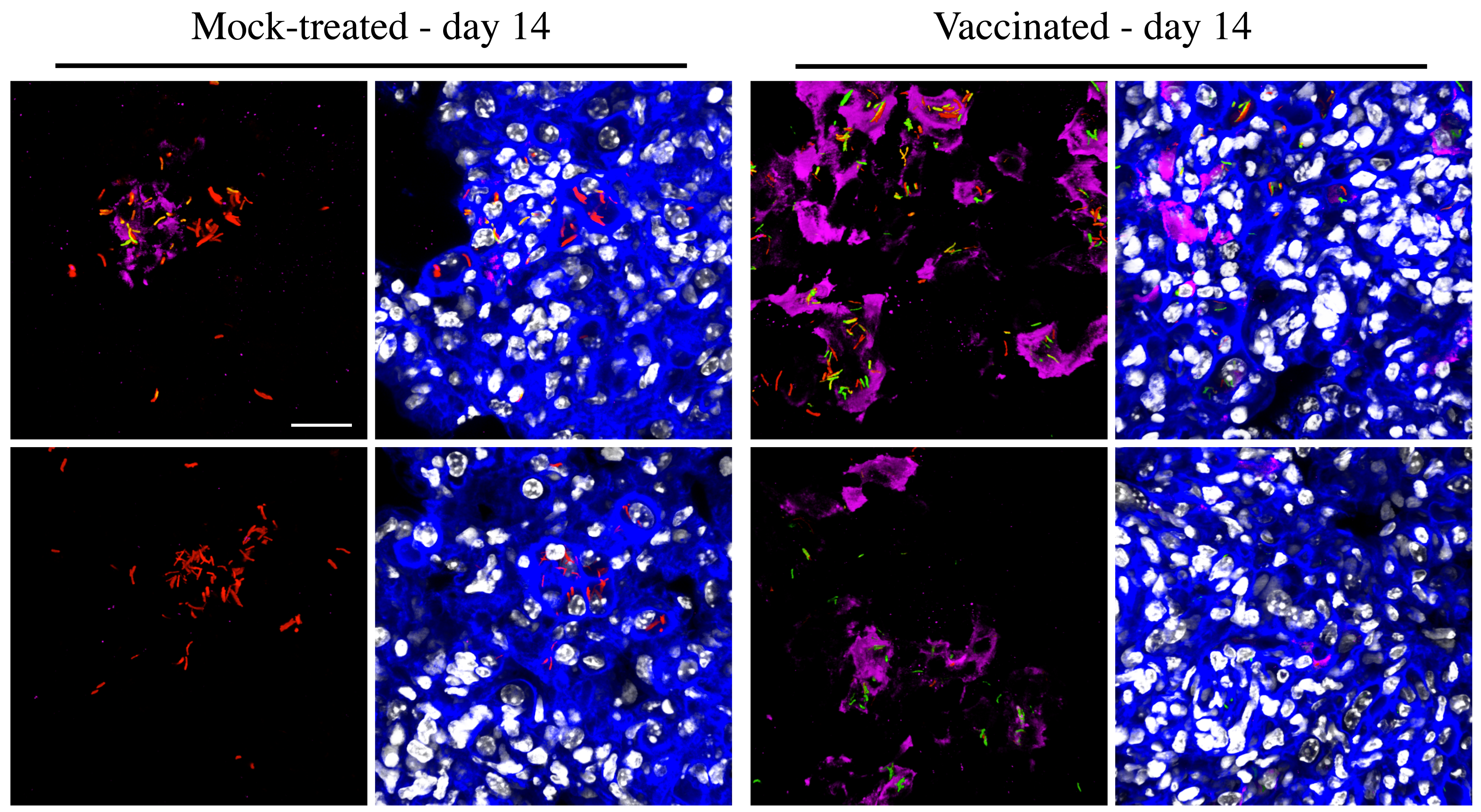

Supplement: Figure S2 — iNOS-positive regions are more frequent in Mtb-infected vaccinated mice lung tissues at day 14 post-challenge. Erdman(hspX′::GFP, smyc′::mCherry) was inoculated into vaccinated or mock-treated C57BL/6J WT mice for 14 days. Two sets of 3D confocal images are shown for each treatment condition, with all bacteria marked in red (smyc′::mCherry), reporter signal shown in green (hspX′::GFP), iNOS stained in magenta, nuclei marked in grayscale (DAPI), and phalloidin staining of f-actin shown in blue. Scale bar 20 µm. (TIF) [file ppat.1004394.s002.tif]
